# Correction: Community health workers experiences and perceptions of working during the COVID-19 pandemic in Lagos, Nigeria—A qualitative study

**DOI:** 10.1371/journal.pone.0315227

**Published:** 2024-12-04

**Authors:** The PLOS ONE Staff

Table 2 is formatted incorrectly. Please see the correct Table 2 here.

**Table 2:** Summary of themes and subthemes.

THEME	SUBTHEMES	CODES	FREQUENCY OF CODES BY PARTICIPANTS
1. Influences on ability to undertake COVID-19 Role	a) Trust	Trust with Community	8
	b) CHW Knowledge about COVID-19	Participant COVID Knowledge	15
	c) Exhaustion	Draining	11
	d) Public misconceptions about COVID-19	Stigma	4
	e) Delayed Access to Care	Misconceptions	10
		Fears of being diagnosed	7
		Fears of getting infected	3
	f) Lack of Transportation	Transportation	1
2. Influences on willingness to work in COVID-19 Role	a) CHW perceptions of COVID-19	Disruptive	5
		Low risk	11
		Threat	8
		Terrible	10
		Adaptation	4
	b) CHW perceptions of Government COVID-19 Response	Government	14
	c) CHW attitude to responsibility for COVID-19 risk at work	Uncertainty	6
		Positive outlook	2
		Personal responsibility	10
	d) Commitment to Their Role	Sense of duty and responsibility	14
		Enjoyment	11
	e) Faith	Faith	12
3. Suggested Improvements	a) Financial Incentives	Financial	11
	b) PPE	PPE	10
	c) Transportation	Transportation	4
	d) More Staff	More Staff	1

The publisher apologizes for the error.
