# Supplementary material for: Community health workers experiences and perceptions of working during the COVID-19 pandemic in Lagos, Nigeria—A qualitative study
Source: PLoS One. 2022 Mar 8;17(3):e0265092. doi: 10.1371/journal.pone.0265092 (PMC8903241; doi:10.1371/journal.pone.0265092)
Supplement: S1 Fig — (PDF) [file pone.0265092.s001.pdf]

Interview Topic Guide

Community Health Workers experiences and perceptions of working during the COVID-19 pandemic in Lagos, Nigeria - A Qualitative Study

Introduction

**Introduction of the Researcher**	Hello, my name is ZO (principal researcher), and I am a medical student at the University of Birmingham, UK. Thank you for choosing to take part in this study. I will be interviewing you today. Before we start the interview, I will briefly explain the study and answer any questions you may have.<br><br>May I ask your name? Is it okay to call you by this?
**Study aims and Purpose**	● This study aims to explore Community Health Workers perceptions and experiences towards the COVID-19 pandemic in Lagos, Nigeria.<br>● This study may help understand how to improve people's response to future pandemic and disease guidance.<br>● Any study results will be sent to TO (local researcher) who will forward the results to yourself and other interested individuals in Lagos, Nigeria.
**Data Collection**	● The interview should take between 30 and 60 minutes.<br>● During the interview I will be asking you about your experiences, thoughts and opinions about the COVID-19 pandemic in Lagos, Nigeria.<br>● With your permission, the interview will be video- and audio-recorded over Zoom.<br>● The interview will be transcribed (written out on a computer) so that the conversation can be analysed.
**Confidentiality**	● Everything said during this interview will be completely confidential.<br>● Anonymised quotations from the interview may be used in our research paper. However, you will not be identifiable in any way.
**Questions**	● Before we continue, do you have any questions?<br>● You may stop the interview at any point to ask a question.<br>● If there is anything you don't wish to discuss we can move on to the next question
**Consent**	● Before we start the interview, I will need to obtain your verbal consent to take part in this study<br>● (Researcher to go through and complete the Participant Consent Form verbally).<br>● If at any point in the interview you would like to stop or pause for a break, please let me know.

<table>
<tr><td>Participant Demographics</td><td>I am now going to complete a form with some background information about you. This will allow us to get some context about the results we get from this study.</td></tr>
<tr><td>Permission to start audio recording</td><td>Thank you. We are now ready to start the interview. Do you have any last-minute questions you would like to ask before we start? (If yes, answer questions accordingly. If no, continue) Are you happy for me to start the audio recording?</td></tr>
</table>

<u>Main body of the interview</u>

<table>
<tr><th>Topic</th><th>Questions and Probes</th></tr>
<tr><td>Knowledge and understanding of COVID-19</td><td>You’re a *insert job role* what was your role like before COVID-19?
What type of community healthcare do you provide?
What do you know about COVID-19? Probe: What caused COVID-19?
Probe: Where did it come from?
Who is more at risk of getting severely ill from COVID-19?</td></tr>
<tr><td>Perceptions/Awareness of the COVID-19 issue in Lagos and Nigeria as a whole</td><td>Can you tell me about the COVID-19 situation in Lagos? What about the rest of Nigeria?
How do you feel about this?
Can you tell me about any adverts or news articles you have seen regarding controlling the spread of COVID-19?
Probe: handwashing, wearing face masks, social distancing?
Where do you get your information regarding COVID-19 from?</td></tr>
<tr><td>Personal and Societal Attitudes to COVID-19</td><td>How do you feel about COVID-19?
What do you think other members of society think about COVID-19? More specifically, in Nigeria?
How would you feel if you were diagnosed with COVID-19?
Did you feel differently about COVID-19 compared to other disease outbreaks? Probe: such as Ebola?</td></tr>
<tr><td>Experiences Working in Lagos during COVID-19/ Management of COVID-19 in the frontline</td><td>What has your experience been of COVID-19?
What was it like working as a Community Health Worker in Lagos during COVID-19?
Have you come into contact with a person who has COVID-19 in Lagos?
Probe: If yes
Where did you come into contact with this person?
Probe: Was it through your job or personal life?
Probe: When did this happen?
Could you tell me how this experience made you feel?
How did you help this person?
Probe: If no, how did this change your attitude towards COVID-</td></tr>
</table>

<table>
<tr><td></td><td>19?

What made you continue to work as a Community Health Worker during COVID-19?
Did you have any worries working during COVID-19?
If yes, what were they?
If no, why not?
How did your role as a Community Health Worker change during the pandemic?
Can you tell me how you were prepared for working during COVID-19?
Probe: Did you receive any training e.g. for infection control? Follow up: Can you tell me more about this?
What protection were you given whilst working during COVID-19?
Probe: How did this protection or lack of protection make you feel?
Probe: Did it make you feel safe?
Probe: Were you given adequate PPE such as masks, gloves, aprons, hand sanitisers?
How supported did you feel whilst working in the pandemic?
</td></tr>
<tr><td>Management of COVID-19 in Lagos, Nigeria</td><td>
Who do you feel is responsible for controlling and managing the spread of COVID-19? Probe: Ask about the role of the government and health professionals
Who were the main authority figures managing the pandemic in Nigeria?
What measures were put in place to control COVID-19?
How do you feel about how the Nigerian government has managed COVID-19?
Probe: What do you think are the appropriate steps needed to control the spread of COVID-19 in Lagos? And were they taken?
How well do you think the average Lagos citizen followed the rules set by the government during the pandemic?
Probe: What do you think made people follow the rules?
Probe: What do you think made people not follow the rules?
How did your knowledge of COVID-19 affect whether you followed the rules?
Probe: Do you think if you knew more or less information, this would’ve affected your decision to follow the rules?
What do you think has been done well or worked well in Lagos?
What do you think could’ve been done better to control COVID-19 in Lagos?
Is there anything that could be done to better support community health workers during the pandemic?
</td></tr>
</table>

<u>Closing of the interview</u>

- That brings us to the end of the interview.
- Is there anything else you’d like to add?
- Do you have any further questions?

- Remind the participant that everything discussed is confidential and they won't be identifiable. Also remind them that they are able to withdraw from the study up to 5 days after the interview.
- If the participant was distressed during the interview, signpost them to TO (local researcher).

Thank you very much for your time and for taking part in this study.
