# Supplementary material for: Community health workers experiences and perceptions of working during the COVID-19 pandemic in Lagos, Nigeria—A qualitative study
Source: PLoS One. 2022 Mar 8;17(3):e0265092. doi: 10.1371/journal.pone.0265092 (PMC8903241; doi:10.1371/journal.pone.0265092)
Supplement: Attachment — Submitted filename: Response to Reviewers.docx [file pone.0265092.s003.docx]

| **Reviewer** | **Reviewer comment** | **Author response** |
| --- | --- | --- |
|  | Please ensure that your manuscript meets PLOS ONE's style requirements, including those for file naming. The PLOS ONE style templates can be found at  <https://journals.plos.org/plosone/s/file?id=wjVg/PLOSOne_formatting_sample_main_body.pdf>  and  <https://journals.plos.org/plosone/s/file?id=ba62/PLOSOne_formatting_sample_title_authors_affiliations.pdf> | The manuscript has been reformatted using these links. This included:   - Reformatting the headings using Heading 1,2 and 3 - Adding indents to paragraphs - Changed manuscript font to Arial - Renamed supporting files |
|  | Please amend your current ethics statement to address the following concerns:  a) Did participants provide their written or verbal informed consent to participate in this study?  b) If consent was verbal, please explain i) why written consent was not obtained, ii) how you documented participant consent, and iii) whether the ethics committees/IRB approved this consent procedure. | Verbal consent was documented by video and audio recording the consent discussion which was uploaded to a secure database. Verbal consent was used due to the practical difficulties of UK-based researchers obtaining written consent from participants based in Nigeria. This was approved by both the University of Birmingham Internal Ethics Review Committee (Reference: IREC2020/1760475) and Lagos University Teaching Hospital Health Research Ethics Committee (Reference: ADM/DCST/HREC/APP/4054). |
|  | Please include a complete copy of PLOS’ questionnaire on inclusivity in global research in your revised manuscript. Our policy for research in this area aims to improve transparency in the reporting of research performed outside of researchers’ own country or community. The policy applies to researchers who have travelled to a different country to conduct research, research with Indigenous populations or their lands, and research on cultural artefacts. The questionnaire can also be requested at the journal’s discretion for any other submissions, even if these conditions are not met.  Please find more information on the policy and a link to download a blank copy of the questionnaire here: <https://journals.plos.org/plosone/s/best-practices-in-research-reporting> . Please upload a completed version of your questionnaire as Supporting Information when you resubmit your manuscript. | We have completed this form and have submitted it as S2 Fig and cited it in our manuscript. (Line 372-374) |
|  | We note that you have indicated that data from this study are available upon request. PLOS only allows data to be available upon request if there are legal or ethical restrictions on sharing data publicly. For more information on unacceptable data access restrictions, please see <http://journals.plos.org/plosone/s/data-availability#loc-unacceptable-data-access-restrictions>. | The data that support the findings of this study are not publicly available due to them containing information that could compromise research participant privacy. The authors did not seek ethical permission from the participants, nor the ethics committee, for the data to be used for anything other than this particular research study. The authors therefore do not have explicit permission for data sharing, re-analysis nor future studies and so would be inappropriate and unethical to make them available in the public domain. While anonymised, the data contains potentially identifying patient information. However, qualified individuals can direct queries by contacting Dr Ruth Riley ([r.riley@bham.ac.uk](mailto:r.riley@bham.ac.uk)) - chair of the University of Birmingham BMedSci Intercalation Internal Ethics Review Committee. |
|  | In your revised cover letter, please address the following prompts:  a) If there are ethical or legal restrictions on sharing a de-identified data set, please explain them in detail (e.g., data contain potentially sensitive information, data are owned by a third-party organization, etc.) and who has imposed them (e.g., an ethics committee). Please also provide contact information for a data access committee, ethics committee, or other institutional body to which data requests may be sent.  b) If there are no restrictions, please upload the minimal anonymized data set necessary to replicate your study findings as either Supporting Information files or to a stable, public repository and provide us with the relevant URLs, DOIs, or accession numbers. For a list of acceptable repositories, please see <http://journals.plos.org/plosone/s/data-availability#loc-recommended-repositories>.  We will update your Data Availability statement on your behalf to reflect the information you provide. | Thank you for highlighting this point. We have added the following statement to the revised cover letter.  “The data that support the findings of this study are not publicly available due to them containing information that could compromise research participant privacy. The authors did not seek ethical permission from the participants, nor the ethics committee, for the data to be used for anything other than this particular research study. The authors therefore do not have explicit permission for data sharing, re-analysis nor future studies and so would be inappropriate and unethical to make them available in the public domain. While anonymised, the data contains potentially identifying patient information. However, qualified individuals can direct queries by contacting Dr Ruth Riley ([r.riley@bham.ac.uk](mailto:r.riley@bham.ac.uk)) - chair of the University of Birmingham BMedSci Intercalation Internal Ethics Review Committee.” |
|  | Please include captions for your Supporting Information files at the end of your manuscript, and update any in-text citations to match accordingly. Please see our Supporting Information guidelines for more information: <http://journals.plos.org/plosone/s/supporting-information> | Thank you for highlighting this point.  We have amended the manuscript to list the supporting information files at the end of the manuscript. (Line 1316-1318) |
|  | Please review your reference list to ensure that it is complete and correct. If you have cited papers that have been retracted, please include the rationale for doing so in the manuscript text, or remove these references and replace them with relevant current references. Any changes to the reference list should be mentioned in the rebuttal letter that accompanies your revised manuscript. If you need to cite a retracted article, indicate the article’s retracted status in the References list and also include a citation and full reference for the retraction notice. | Thank you for highlighting this point. We have gone through the reference list to check for retracted papers and none were found. The reference list has slightly changed due to adding in and removing text based on the reviewers comments. These changes have been listed below (Line 1052-1314)  **The following changes to the original reference list were made:**  References 2,5-9 were removed due to reviewer comment 7.  References 44-50 were removed due to no need to reference the different cadres of CHWs in this section.  Reference 57 was removed because we have already referenced the use of reflexivity process with number 56 (now 43)  References 71 and 72 were removed due to reviewer comment 18.  **New references added/changed:**  Number 7 and 8 has been added to reference the training that CHW receive in response to reviewer comment 1.  Number 9 the link has been changed to hyperlink to the full article rather than the abstract.  Number 11 the link has been changed to hyperlink to the full article.  Number 31 was added to help explain why a generic study design was chosen.  Number 37 the two authors have been added to the authors list and the link has been changed to hyperlink to the full article. |
| Additional Editor Comments: | | |
|  | There are minor grammatical errors throughout the manuscript. English language correction is deemed necessary. | All reviewers have read through the manuscript and corrected grammatical errors found. |
| 1 | 1.       Line 83: CHW – What training do they get? | Thank you for highlighting this point.  We have amended the manuscript to describe the training received by CHWs in the study. (Line 97-106) |
|  | 2.   Lines 112-121: If a focus group qual study was undertaken in 2020 (Mayfield-Johnson et al) then why is this study being carried out? It would be good to justify the need for this study in a bit more detail. Were there any gaps in the findings from previous studies that led to the research questions/ aims for this study? What would this study add to the current evidence? | Thank you for highlighting this point.  We noticed an error in this section of the manuscript which stated that the focus group by Mayfield-Johnson was conducted in Nigeria when it was conducted in the United States. We have amended the manuscript to correct this mistake and to better describe the gap in the literature and need for this study. (Line 229-235) |
|  | 3.   Line 131: What is a generic qualitative study? Do you mean it’s not aligned to any particular epistemological/philosophical school of thought? Why was this study design chosen? | We have explained why this study design was chosen. (Line 290-295) |
|  | 4. Line 144: Why was purposive and snowball sampling used? Any limitations to this approach? | Thank you for highlighting this point.  We have amended the manuscript to explain why purposive and snowball sampling was used and the limitations of this approach. (Line 306-310, 1009-1011) |
|  | 5. Line 165: “experiences working during the pandemic……” Typo – should say “experiences of working during…..” | Thank you for highlighting this point.  We have amended the manuscript to say “experiences of working during…..” (Line 378) |
| 2 | 6. Abstract Background: reduce some statements and add the aim of the study as a last sentence to the background. | Thank you for highlighting this point.  We have reduced the background and added the aim of the study to the end of the background (Line 25-29) |
|  | 7. Introduction Information on paragraph 1 and 2, lines 66-78 may not be necessary. I suggest that the authors focus on the global role of CHWs, their role in Africa and the impact and challenges they are facing in the face of the pandemic. | Thank you for highlighting this point.  We have amended the introduction by reducing the content in the first two paragraphs and giving more focus to CHWs and their role. (Line 87-90) |
|  | 8. Line 88; “who” should be deleted. | On review this sentence does not make sense if ‘who’ is removed, as it refers to the communities served by the CHWs. (Line 110) |
|  | 9. Statement for lines 98 and 99 should be paraphrased. | Thank you for highlighting this point.  We have amended these lines to make them more succinct (Line 165-166) |
|  | 10. Materials and methods Data analysis What type of coding frame did you use? Was it hierarchical or flat coding frame? There is need to state the type of coding frame that was used for the analysis. | We have amended the manuscript to state that a hierarchical coding frame was used. (Line 404) |
|  | 11. In your methodology, you made mention that you used inductive and deductive method of coding which is unclear. It is assumed that you start coding your data from the scratch when you use inductive method of coding and all your codes are generated from your data. Also, if iterative process was used in analyzing your data, from my understanding, it means that you used inductive coding while analyzing data and at the same time refining the topics for further interview? How did you combine both inductive and deductive method of coding in analyzing your data since deductive coding has to do with a predefined set of codes that are used in data analysis? | Thank you for highlighting this point. We have amended the manuscript to state that only inductive analysis was used as data was coded from scratch (Line 393,424) |
|  | 12. In your methodology, you stated procedures that you used in carrying out the analysis using the thematic method of analysis. I think you should state that seven stages are involved in the frame work method of analysis and explain what was done at each stage for easy comprehension: Transcription; familiarization with the interview; coding; developing a working analytical framework; applying the analytical framework; charting data into the framework matrix; and interpreting the data. | Thank you for highlighting this point.  We have amended the data analysis section of the manuscript to explain each stage of the framework method (Line 394-421) |
|  | 13. Reflexive process was used to eliminate bias, inductive method which you stated as part of your coding methodology is also a good method in eliminating bias during data analysis. It will be good to add it as part of the method that was used to eliminate bias in your study. | Thank you for highlighting this point.  We have amended the manuscript to state that the inductive process helped to eliminate bias (Line 424-426) |
|  | 14. Ethics Statement Under the ethics statement, it will be nice to state that verbal consent was obtained from all participants after explaining to them the content of the research before commencement of the interview for each participant. | Thank you for highlighting this point.  We have amended the ethics statement to state the process of obtaining verbal consent from participants (Line 432-433) |
|  | 15. Results Table 2 1. Influences on ability to undertake COVID-19 Role a) Trust b) CHW Knowledge about COVID-19 Looking closely at table 2 theme 1; could it be possible that subthemes c) Exhaustion d) Stigma e) Public misconceptions about COVID-19 f) Delayed Access to Care g) Lack of Transportation could be under a new theme tagged “Challenges faced by CHWs”. I think that those challenges that were mentioned was not what influenced the CHWs to take up the COVID-19 role, rather, these were the hitches they encountered while carrying out their duties. | Thank you for your suggested new theme name. however, after careful consideration we have decided to stick with the original theme title as two of the sub themes are facilitators to participants roles not challenges. (Line 485-497) |
|  | 16. Table 2, already having the themes and subthemes; needs to be further developed by adding columns for “codes” and the “frequency of codes by participants” to further elaborate on the findings of your analysis and for clarity. | We have added further columns to include “codes” and the “frequency of codes by participants”.  (Line 485-497) |
|  | 17. Discussion Paraphrase the statement; lines 534 and 535 "Considering the inevitability of these challenges in any disease outbreak is important due to their sudden and unfamiliar nature." | Thank you for highlighting this point.  We have paraphrased these lines to say “However, due to the sudden nature of disease outbreaks, such challenges can be expected.” (Line 883-884) |
|  | 18. Strengths and Limitations Lines 599 and 600; The majority of participants were female; however, this reflects CHWs being a female dominant profession. The statement needs to be paraphrased. The participants for this study were selected based on purposive sampling, I don’t think is safe to assume that the majority of CHWs in Lagos State or under the LGAs where the study was conducted are female workers except where such data is available. | Thank you for highlighting this point.  We have amended the manuscript to say “The majority of participants were female, however there did not seem to be any gender-based differences in findings” (Line 1004-1005) |
|  | 19. Reaching data saturation in a qualitative study is import to ensure that justice is done with regards to a particular topic. As part of the limitation of this study, you stated that data saturation was not reached due to the fact that new information was generated during the last interview that was conducted. As part of your recommendation, it is good to state the need for further studies in this regard to explore all options which the current study was not able to cover since data saturation was not reached. | Thank you for highlighting this point.  We have amended the manuscript to say “Therefore, further studies are needed to further explore the experiences and perceptions of CHWs which the current study was not able to cover.” (Line 1021-1023) |
